# Composition, Diversity and Abundance of Gut Microbiome in Prediabetes and Type 2 Diabetes

**DOI:** 10.15436/2376-0949.15.031

**Published:** 2015-12-26

**Authors:** Stacey M Lambeth, Trechelle Carson, Janae Lowe, Thiruvarangan Ramaraj, Jonathan W. Leff, Li Luo, Callum J Bell, Vallabh O Shah

**Affiliations:** 1 University of New Mexico Health Sciences Center, Albuquerque, NM, 87131, USA; 2 National Center for Genome Resources, Santa Fe, NM, 87505, USA; 3 University of Colorado at Boulder, Boulder, CO, 80309, USA

**Keywords:** Prediabetes, Diabetes, Gut microbiome, Bacterial diversity

## Abstract

Association between type 2 diabetes (T2DM) and compositional changes in the gut micro biota is established, however little is known about the dysbiosis in early stages of Prediabetes (preDM). The purpose of this investigation is to elucidate the characteristics of the gut micro biome in preDM and T2DM, compared to Non-Diabetic (nonDM) subjects.

Forty nine subjects were recruited for this study, 15 nonDM, 20 preDM and 14 T2DM. Bacterial community composition and diversity were investigated in fecal DNA samples using Illumina sequencing of the V4 region within the 16S rRNA gene.

The five most abundant phyla identified were: *Bacteroidetes, Firmicutes, Proteobacteria, Verrucomicrobia*, and *Actinobacteria*. Class *Chloracido* bacteria was increased in preDM compared to T2DM (p = 0.04). An unknown genus from family *Pseudonocardiaceae* was significantly present in preDM group compared to the others (p = 0.04). Genus *Collinsella*, and an unknown genus belonging to family *Enterobacteriaceae* were both found to be significantly increased in T2DM compared to the other groups (Collinsella, and p = 0.03, *Enterobacteriaceae* genus p = 0.02). PERMANOVA and Mantel tests performed did not reveal a relationship between overall composition and diagnosis group or HbA1C level.

This study identified dysbiosis associated with both preDM and T2DM, specifically at the class and genus levels suggesting that earlier treatment in preDM could possibly have an impact on the intestinal micro flora transitioning to T2DM.

## Introduction

### Diabetes & prediabetes

Diabetes mellitus is a group of diseases marked by disordered insulin resulting in elevated blood glucose levels^[1]^. This group of diseases affects an estimated 9% of the global population^[2]^ and approximately 9.3% or 29.1 million people in the United States^[1]^. In 2012, it was estimated to cost the US $245 billion, accounting for both direct and indirect costs^[1]^. Type 2 Diabetes Mellitus (T2DM) constitutes at least 90% of diabetes cases in the adult population^[1]^. This condition is considered to be a heterogeneous and multi factorial disease, influenced by both environmental and genetic factors^[1]^. T2DM continues to be a leading cause of renal failure, non-traumatic limb amputations, and blindness among adults^[1]^. It is a major contributor to both cardiovascular disease and stroke, and was reported as the seventh leading cause of death in the US in 2010^[1]^.

Prediabetes (preDM) is an intermediate state between non-diabetic and diabetic plasma glucose levels^[3]^. Specifically, it is defined as: fasting glucose levels 100-125 mg/dl, plasma glucose 140-199 mg/dl on two-hour Oral Glucose Tolerance Test (OGTT), or Glycated Hemoglobin (HbA1C) level between 5.7 and 6.4%^[3]^. In 2012 an estimated 37% of US adults, effectively 86 million people, qualify as PreDM^[1]^. The preDM state is associated with obesity, hypertension, and hypercholesterolemia and is considered a risk factor for both cardiovascular disease and T2DM[^[3]^. Those with HbA1C levels within the preDM range (5.7-6.4%) have an increased relative risk of developing T2DM in 5-years compared to those with normal levels, and the higher the HbA1C, the greater the risk^[4]^. Prevention of the transition to T2DM has been proven successful with weight loss, exercise programs, and pharmacologic agents such as Metformin^[5]^. Thus far, preDM is considered a multi factorial condition caused by genetic predisposition, increased insulin demand, and decreased pancreatic beta-cell mass^[6]^. The patho physiology of the preDM state, and the mechanisms underlying the progression to T2DM are important for the development of further interventions to alleviate the burden of T2DM.

### Human microbiome

In recent years, there has been increasing interest in the microbes that inhabit the human body, or the ‘human micro biome’^[7]^. This micro biome involves approximately 100 trillion microbial organisms that inhabit and are believed to influence important physiological human processes^[7,8]^. These organisms are thought to interact with their environment through quorum sensing, nutrient production, signaling pathway modulation, and gene transfer^[8]^. Interestingly, the human micro biome has been shown to represent a pliable meta genome that varies from individual-to-individual, disease-to disease, and among anatomical locations within each individual^[8,9]^. Characterization of what is considered normal flora, has been undertaken for certain anatomical locations such as the skin, mouth, nasal cavities, vagina, and gastrointestinal tract^[7,9]^. Current micro biome techniques are based on sequencing of the bacterial 16S ribosomal RNA gene, phylogenetically identifying it, and quantifying the number of genes present^[7]^. The micro biome is currently being described in terms of richness and diversity, composition, and functionality^[7-12]^. Based on available research, a ‘normal’ or ‘healthy’ gut micro biome is composed of the bacterial *phylaFirmicutes* and *Bacteroidetes* (>90%), followed by *Actinobacteria* and *Verrucomicrobia*; it contains a very small (0.1%) amount of pathogenic and opportunistic species^[8-10]^. Based on a study of Danish participants^[11]^, those who had increased numbers of bacterial genes (richness) exhibited healthier phenotypes and also had the following intestinal micro biome characteristics: presence of methanogenic/acetogenic communities, increased butyrate-producing bacteria, increased ratio of *Akkermansia: Ruminococcus torque/gnavus*, increased potential for hydrogen production, decreased potential for hydrogen sulfide production, and reduced number of *Campylobacter* and *Shigella* genera. Based on available research, the various functions of the intestinal micro biome are preserved despite a wide variety of species composition^[9]^. Function is implied by characteristics of the species present, by meta genomic techniques that identify genes involved in functional pathways rather than by phylogeny, and by direct measurement of the byproducts of bacterial metabolism^[9,11,13]^. Functional pathways being studied include nutrient metabolism and harvest, immuno modulation, and inflammation^[8,10]^.

In patients with both local and systemic disease processes, an alteration in the normal micro biota, or dysbiosis, is apparent^[8]^. Dysbiosis has been implicated in either the cause orthe effect of localized disease such as dental caries, bacterial vaginosis, and inflammatory bowel disease; and systemic conditions such as obesity or allergies^[8]^. The effect of intestinal micro biota on whole-body metabolism and obesity began with studies in mice and quickly expanded to include humans^[8]^. Murine studies revealed a relative increase in *phylaFirmicutes* compared to *Bacteroidetesin* the intestines of obese mice^[12]^, this was confirmed in some human studies^[14]^, and not in others^[11]^. When examining the function of the gut micro biome, studies have suggested an overall increased capacity for energy harvest from the diet in obese individuals^[12,15]^.

The interconnection between gut micro biota and metabolic disease initiated interest into the relationship between gut micro biota and T2DM. One study demonstrated that compositional changes in the intestinal micro biota were associated with T2DM compared to non-diabetic controls^[16]^. This study demonstrated a significantly lower abundance of the phylum Firmicutesand class Clostridia, meanwhile a significantly higher abundance of class *BetaProteobacteria*^[16]^. They also found that the ratio *of PhylaBacteroidetes: Firmicutes* was increased in T2DM and positively correlated with increasing plasma glucose on OGTT^[16]^. A study conducted on 345 Chinese individuals^[17]^ found no difference in micro biome diversity between T2DM and non-DM patients, but did find differences in composition/ function including increased: butyrate-producing bacteria, opportunistic pathogens, and species with potential for sulfate-reduction and mucin-degradation. They also identified groups of genes that were found to co-exist and were enriched in either T2DM or control subjects; for example, 337 genes belonging to the species *Akkermansia muciniphila* were enriched in T2DM, whereas 273 genes belonging to *Haemophilus parainfluenzae* were enriched in control subjects^[17]^. There is an increasing body of knowledge on the subject of intestinal dysbiosis in T2DM; however, it is unknown whether these differences occur early in preDM patients, and whether or not they help to mediate the onset of T2DM.

A recent study looked at the intestinal micro biota of Chinese individuals who were categorized into three groups based on their Fasting Plasma Glucose (FPG) level^[18]^. This study revealed higher levels of class Clostridia and lower level of class *Bacteroidia* in T2DM compared to preDM and normal groups, genus Streptococcus was most abundant in the normal group and decreased in PreDM and further in the T2DM group, levels of genera *Prevotella* and *Megamonas* were higher than in the normal group^[18]^. The study presented in this article aims to answer a similar question: what is the composition of the gut micro biome belonging to preDM patients? Does it have similarities to those with T2DM? Does it differ significantly from non-diabetics?

## Materials and Methods

### Subjects

The University of New Mexico Health Sciences Center Human Research Review Committee Institutional Review Board approved this study and all participants rendered written informed consent and received $25 for their participation. A preDM cohort of 200 participants was initially created in 2012-2014 from established patients attending a primary care clinic of the University of New Mexico Health Sciences Center. For this pilot study a total of 71 willing and available participants were recalled from a Family Practice Clinic in Albuquerque, NM. Information regarding pertinent medical history, demographics, current medications, diet, alcohol and tobacco use was obtained by means of a survey questionnaire administered by a member of the research team. Height, weight, waist circumference, and blood pressure were measured according to standard procedure. Patients who were acutely ill or actively taking antibiotics were excluded. A fasting blood sample was obtained from each subject by venipuncture for the determination of HbA1C, glucose, creatinine, albumin, total protein, uric acid, and lipids. HbA1C values, according to the ADA classification system^[3]^, were used to categorize subjects as nonDM (< 5.7 %), preDM (5.7 - 6.4%), or T2DM (> 6.5%). All subjects provided a urine sample for the measurement of Urine Creatinine (UACR) and micro albumin as well as a stool sample for the study of intestinal micro biome. Stool samples were handled as previously described^[19]^; samples were frozen at -20°C for up to 24h after voiding and then frozen at -80°C until DNA extraction. Clinical chemistry measurements were performed at the Tricore Reference Laboratories, Albuquerque, NM using clinical diagnostic assays certified by The Clinical Laboratory Improvement Amendments (CLIA) of the Centers for Medicare and Medicaid Services.

### DNA Extraction, PCR amplification, and sequencing

DNA was extracted individually from all patients' stool samples using QiaAMP mini stool kit (Qiagen, Valencia, CA, USA). To assess the composition and diversity of the patients' gut bacterial communities, we were able to use only 49 samples with intact and good quantity of DNA to conduct high-throughput sequencing of the V4 region of the 16S rRNA gene^[20]^. PCR amplification was performed on this region in triplicate using the 515f/806r primer pair with unique 12 bp barcodes specific to individual samples and combined the resulting product for each sample. PCR product was quantified using the Pico Green dsDNA assay, and the samples' bar-coded amplicons were combined in equimolar concentrations. Sequencing was performed on an Illumina MiSeq instrument to produce 150 bp sequences at the University of Colorado at Boulder.

### Gut microbial community composition and diversity

Quality filtering, assignment of sequences to individual samples based on their barcodes, And Operational Taxonomic Units (OTU) clustering was performed using the QIIME (Quantitative Insights into Microbial Ecology) v.1.7.0 pipeline^[21]^. The closed reference-based OTU picking protocol was used along with other default parameters^[22]^. In this approach sequence reads for each sample were clustered against a reference sequence collection and sequences < 97% similar to any reference sequence were excluded from downstream analyses. This approach implements reference based clustering using the UCLUST^[23]^ algorithm and the Green genes^[24]^ reference database that covers most of the organisms that are typically present in the human gut micro biome. High percentages (80-90%) of reads were classified using this approach. Because we obtained a variable number of sequences per sample ranging from 14,916 to 36,631 (Supplemental Table 1 for yield per sample after initial processing and closed-reference OTU picking), the sequence data were rarefied to 14,900 sequences per sample to account for this variation. No samples were lost in this study due to rarefaction. This depth of sampling has been shown to be more than sufficient to make assessments of diversity and community composition diversity patterns across varied treatments^[25]^.

### Statistical analysis

Clinical data was expressed in means plus standard deviations, and differences between the groups were assessed using one-way ANOVA and post-hoc analysis was done by Turkey's honest significance test to find means that are significantly different from each other in normal, preDM and T2DM. For assessment of micro biota, taxa were represented at a particular phylogenetic resolution (phylum, class and genus) that had a relative abundance of at least 0.1% in any of the three groups. The relative abundances were compared across three groups using Kruskal-Wallis rank sum tests^[26]^ and if significant, then pair wise comparison; p values were corrected using False Discovery Rate (FDR) to account for multiple comparisons. Principal Component Analysis (PCA) and Per Mutational Multivariate Analysis Of Variance (PERMANOVA) were used to analyze the relationship between overall micro biome composition and diagnosis group, and dissimilarities between composition and HbA1C were assessed using Mantel tests. Three dissimilarity metrics were used: *Bray-Curtis, unweighted UniFrac*, and *weighted UniFrac*^[27]^. Alpha diversity (within-group diversity) was assessed using Shannon diversity index.

## Results

### Group characteristics

Demographic and clinical characteristic of the cohort (n = 49) is presented in table 1. Briefly, we recruited more female (n = 32) than male participants in the study cohort and more Caucasian white (n = 28) than Hispanics (n = 15) with three Native Americans and four others (participants didn't identify the race or it was not listed). The age distribution was similar across the three clinical groups with mean age of 55.5 ± 13.7 yrs in nonDM, 56.0 ± 11.5 yrs in preDM, and 62.0 ± 10.0 yrs in T2DM. Body Mass Index (BMI) was not statistically different across the groups (means 29.2 ± 4.8, 29.7 ± 5.8, 32.1 ± 7.2 for nonDM, preDM, and T2DM respectively). Interestingly, LDL levels were significantly lower in the T2DM group than in the nonDM group (77.1 15.8 mg/dl and 93.7± 24.3 mg/dl, respectively, p = 0.04), while the preDM group had significantly higher levels (113.9 ± 30.9 mg/dl) than T2DM and controls (p = 0.0027, p = 0.04, respectively). This is easily explained by the fact that 57% of the T2DM patients, as compared to 13% of control and 25% of preDM, were on a HMG-CoA reductase medication at the time, which is used clinically to decrease serum LDL levels and reduce cardiovascular events. The kidney phenotypes including UACR, uric acid, and creatinine were not different among or between the groups. Though nephropathy marked by albuminuria is a well-known complication of T2DM^[28]^, non-difference in the UACR in our population, could be explained by the fact that 36% of T2DM group was taking an ACE inhibitor or angiotensin receptor blocker at the time of interview, which decreases the amount of albumin in the urine^[28]^. All patients participating in micro biome analysis were not actively taking antibiotics, nor had they taken any in the one month prior. Eleven participants reported to be on anti-Gastro Esophageal Reflux Disease (GERD) medication and three reported to be taking probiotics.

### Compositional differences

**Figure 1:**
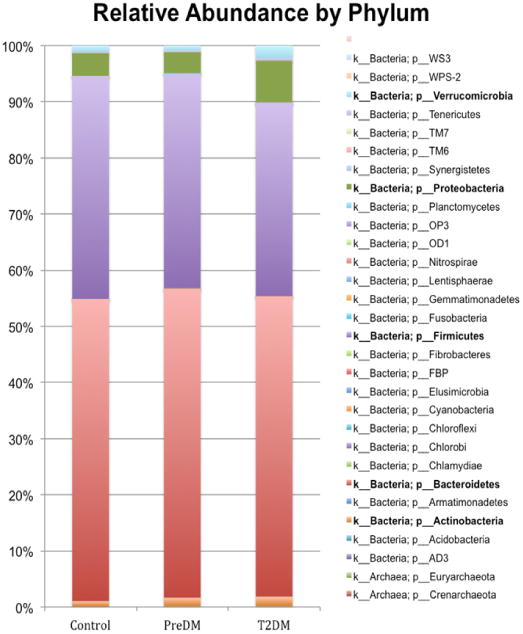
Bacterial composition Using Mean Relative Abundances by Bacterial Phylum. K -indicates kingdom, P - indicates phylum. Bolded names indicate the most abundant phyla.

The output from 49 samples yielded OTU counts ranging from 14,916 to 36,631, with an average of 29,414 for nonDM subjects, 27,438 for preDM, and 28,859 for T2DM (NS). Among these samples there were over 4000 different bacterial species, 440 different genera, 264 families, 90 classes, and 30 phyla. Mean relative abundance and standard deviation are represented for phyla, class, and genera (Supplemental Tables 2a-c). The five most abundant phyla identified were: *Bacteroidetes, Firmicutes, Proteobacteria, Verrucomicrobia, and Actinobacteria* (Figure 1, Supplemental Table 2a) which is consistent with previous findings^[7-11]^. Relative abundances of *Bacteroidetes* and *Firmicutes* were 53.9 and 39.7% respectively in nonDM, 55.0 and 38.2% in preDM, and 53.5 and 34.4% in T2DM (Figure 1). Phylum Synergistetes was significantly increased in T2DM compared to nonDM, however this was nominally significant after FDR correction (Table 2). Three classes out of 90 were identified as significantly different among the groups by Kruskal-Wallis (Table 2). Class *Chloracidobacteria* was increased in preDM compared to T2DM (p = 0.04). Class *Saprospirae* was higher in nonDM versus preDM, but this lost significance with FDR correction; similarly, *Synergistia* was significantly increased in T2DM compared to nonDM before correction. Nine genera out of 440, were identified with some group wise differences (Table 2). An unknown genus from family *Pseudonocardiaceae* was significantly present in PreDM group compared to the others, whom had none detected (p = 0.04). Genus *Collinsella*, and an unknown genus belonging to family *Enterobacteriaceae* were both found to be significantly increased in T2DM compared to the other groups (*Collinsella*, p = 0.03, *Enterobacteriaceae* genus p = 0.02). *Megasphaera* and *Candidatus Solibacter* were increased in preDM compared to the nonDM group, but were not significant after correction; *Lachnospira* and an unknown genus belonging to *Erysipelotrichaceae* were higher in the nonDM group, but also lost significance with correction. Genus *Bulleidia* was present in T2DM while it was absent in the other groups, which lost significance after correction.

**Figure 2:**
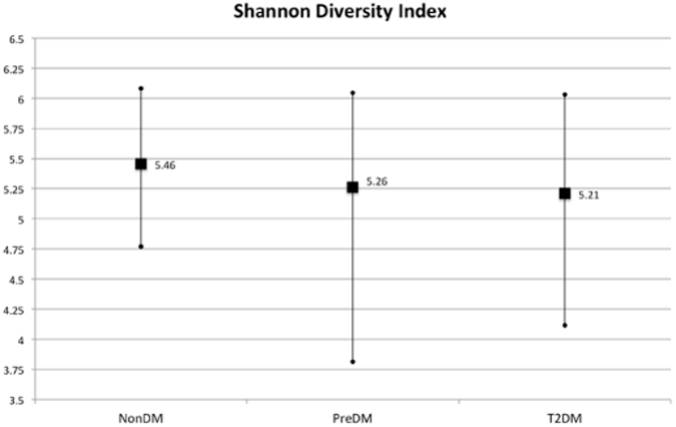
Results of Shannon diversity index. Vertical lines indicate range, squares represent mean values.

Based on PCA and PERMANOVA, a relationship between diagnosis group and micro biome composition were not significant (Supplemental Figure 1, Table 3). Based on Mantel test, we did not find a direct correlation between HbA1C level and dissimilarities in community composition (Table 3). PreDM and T2DM patients had slightly lower Shannon diversity indices, but this was non-significant (Figure 2).

## Discussion

This study evaluated the compositional changes in gut micro biota of normal, preDM and T2DM participants. The baseline characteristics of our study population show a significant difference between and among groups with regard to HbA1C, cholesterol, HDL, and LDL levels; however the remaining clinical parameters were not statistically different. Our results reveal dysbiosis in the gut microbiome of both preDM and T2DM patients in comparison to nonDM patients. We did not find an overall correlation between microbiome composition or diversity and HbA1C level. This indicates that there did not exist a particular pattern of bacterial abundance that associated with either HbA1C or diagnosis group. However, there were many differences found in the individual relative abundances of specific taxa between the three groups. This finding may indicate that there is not a specific gut pattern associated with glucose levels or the diabetic disease state; it may also mean that larger studies needed to see a consistent pattern.

Looking at individual taxa, the preDM group in our study had a preponderance of the class *Chloracidobacteria* and an unknown genus from family *Pseudonocardiaceae. Chloracidobacteria* was belongs to phylum *Acidobacteria*, which is known to inhabit soil globally^[29]^, and has been found in small amounts on leaf salad vegetables^[30]^; however, it is not consistently reported in gut microbiome data. *Pseudonocardiaceae* belongs to phylum *Actinobacteria*, which has been increased in obesity^[31]^, but not consistently.

The T2DM group had higher levels of genus *Collinsella* and an unknown genus belonging to the family *Enterobacteriaceae*. The increase in *Collinsella* in T2DM was a similar finding in^[18]^, and has been associated with symptomatic atherosclerosis in other studies^[32]^. This may be an indication that many of our T2DM subjects had co-morbid symptomatic atherosclerosis, which is expected in a diabetic population^[1]^. The family of *Enterobacteriaceae* contains many gram-negative, pathogenic genera such as: *Escherichia, Klebsiella, Yersinia, Citrobacter, Proteus, Shigella, Salmonella*, and *Serratia. Lipo-polysaccharide* (LPS), which is a cellular-membrane component of such gram-negative bacteria, is increased in both obese and T2DM subjects^[33]^; increased adherence of intestinal Escherichia coli (gram-negative) and a decrease in intestinal *Bifidobacterium* species are associated with increased serum LPS^[34]^. Qin, et al.^[17]^ did find T2DM group with increased levels of Escherichia coli, but not others from the family of *Enterobacteriaceae*. Our results were not analyzed to the species level.

We did not observe differences in the abundances of phyla *Bacteroidetes Firmicutes*, classes *Clostridia* or *Bacteroidia*, nor in genera *Streptococcus, Prevotella*, or *Megamonas* as the previously reported^[18]^. Non-significance in some of our results may have been related to a relatively small sample size or related to a small amount of lost data after rarefaction to 14900 OTUs.

There are many potential confounding factors when it comes to assessing intestinal microbiome. The known association between BMI, obesity and gut microbiome^[11,12,31]^ could have affected the results, though our three groups had mean BMIs, which were not statistically different (Table 1). Diet is a known factor in development of one's intestinal microbiome^[35-37]^, and could have affected our results as well. These participants reported whether they were vegetarian, lactose-free, or gluten-free, but other details of dietary habits were not explored. Diets high in carbohydrates have been associated with a preponderance of genus *Prevotella* and high fat/protein diets have been associated with higher levels of genus *Bacteroides*^[35]^. Medications such as Metformin have been associated with a change in gut microbiome; specifically one study found that there was an increase in *Firmicutes* and decrease in *Bacteroidetes* in patients taking Metformin^[38]^, the patients in this study were not questioned about Metformin specifically at the time of interview, but it can be assumed that some of the T2DM group was taking the medication, and possibly some of the preDM group as well. The effects of probiotics are currently being researched, and so far have reported to have significant effects on metabolism and intestinal mucosal integrity^[39]^. In this study, one patient from each of the diagnosis groups reported taking probiotics.

We report dysbiosis associated with both preDM and T2DM, specifically at the class and genus levels suggesting that earlier treatment in preDM could possibly have an impact on the intestinal micro flora transitioning to T2DM.

## Figures and Tables

**Table 1:** Clinical characteristics of study participants

	NonDM	PreDM	T2DM
N= 49	15	20	14
Gender	67% F 33% M	70%F 30% M	57% F 43% M
Age (Yrs)	55.5 ± 13.7	56.0 ± 11.5	62.0 ± 10.0
BMI (kg/m^2^)	29.2 ± 4.8	29.7 ± 5.8	32.1 ± 7.2
Waist (cm)	96.0 ± 12.8	98.7 ± 20.1	106.7 ± 19.7
**Diabetes Markers**			
Glucose (mg/dL)	92.2 ± 17.8	94.8 ± 14.5*	136.7 ± 32.4*
HbA1c (%)	5.4 ± 0.1	5.9 ± 0.2*	7.9 ± 1.7*
**Kidney Markers**			
UACR	8.0 ± 5.7	43.5 ± 109.0	69.8 ± 131.5
Uric Acid (mg/dL)	4.7 ± 1.1	5.0 ± 1.3	5.1 ± 1.5
Cr (mg/dL)	0.8 ± 0.2	0.84 ± 0.3	0.75 ± 0.3
**Lipid Panel**			
Total Cholesterol (mg/dL)	177.8 ± 29.0	191.6 ± 38.2	160.4 ± 27.7*
HDL (mg/dL)	53.5 ± 18.4	52.4 ± 15.7*	40.6 ± 13.9*
LDL (mg/dL)	93.7 ± 24.3	113.9 ± 30.9*	77.1 ± 15.8*
Triglyceride (mg/dL)	153.4 ± 75.0	126.0 ± 61.8	234.8 ± 187.2*

Mean ± standard deviation, results of one-way ANOVA and pairwise t- tests.N = NonDM, P = PreDM, D=T2DM. UACR= Urine albumin to creatinine ratio.

* Indicates p <0.05

**Table 2:** Relative abundance of taxonomies which demonstrated statistical significance after FDR adjustment. P values are for the narrowest taxonomy listed. Results shown are means, group wise and pairwise p values and FDR-adjusted p values.

Bacterial Taxonomy	Mean Relative Abundance			Raw P value / FDR P value			
	N	P	D	All	N *vs* P	N *vs* D	P *vs* D
*Synergistetes*	0	0.0013%	0.0412%	*0.048	0.230/0.230	*0.032/0.095	0.120/0.181
***Acidobacteria, Chloracidobacteria***	0.0161%	0.0178%	0.0091%	*0.038	0.622/0.622	0.067/0.101	*0.012/*0.037
***Bacteroidetes, Saprospirae***	0.0300%	0.0188%	0.0278%	*0.048	*0.026/0.078	0.723/0.723	0.069/0.103
***Synergistetes, Synergistia***	0.0000%	0.0013%	0.0412%	*0.048	0.230/0.230	*0.032/0.095	0.120/0.181
*Acidobacteria, Solibacteres, Solibacterales, Solibacteraceae, Candidatus Solibacter*	0.0018%	0.0047%	0.0014%	*0.036	*0.041/0.071	1.000/1.000	0.047/0.071
*Actinobacteria, Actinobacteria, Actinomycetales, Pseudonocardiaceae, (unknown genus)*	0.0000%	0.0020%	0.0000%	*0.008	*0.023/*0.042	1.000/1.000	*0.028/*0.042
*Actinobacteria, Coriobacteriia, Coriobacteriales, Coriobacteriaceae, Collinsella*	0.0908%	0.0624%	0.2637%	*0.032	0.546/0.546	*0.025/*0.039	*0.026/*0.039
*Firmicutes, Clostridia, Clostridiales, Lachnospiraceae, Lachnospira*	3.1468%	1.5362%	1.5014%	*0.045	*0.019/0.058	*0.050/0.074	0.648/0.648
*Firmicutes, Clostridia, Clostridiales, Veillonellaceae, Megasphaera*	0.0000%	0.1013%	0.0441%	*0.036	*0.024/0.072	0.334/0.334	0.144/0.216
*Firmicutes, Erysipelotrichi, Erysipelotrichales, Erysipelotrichaceae, (unknown genus)*	0.5217%	0.2507%	0.1486%	*0.030	0.092/0.138	*0.017/0.051	0.141/0.141
*Firmicutes, Erysipelotrichi, Erysipelotrichales, Erysipelotrichaceae, Bulleidia*	0.0000%	0.0000%	0.0038%	*0.020	1.00/1.00	0.069/0.104	*0.036/0.104
*Proteobacteria, Gammaproteobacteria, Enterobacteriales, Enterobacteriaceae, (unknown genus)*	0.2465%	0.4275%	3.7804%	*0.016	0.786/0.786	*0.010/*0.025	*0.017/*0.025

N=NonDM, P=PreDM, D=T2DM

* Indicates p <0.05

**Table 3:** Results of PERMANOVA and Mantel testscalculated using Bray-Curtis, unweighted UniFrac, and eighted UniFrac. p value of > 0.05 represents diabetes diagnosis and A1C level and were not significant.

Correction	PERMNOVA	MANTEL	
	*Diagnosis*	*A1C value*	
	p value	p value	rho
Bray-Curtis (BC)	0.1	0.2	0.07
Unweighted UniFrac (UWUF)	0.2	0.2	0.07
Weighted UniFrac (WUF)	0.2	0.2	0.05
